# Transposable elements contribute to the genomic response to insecticides in *Drosophila melanogaster*

**DOI:** 10.1098/rstb.2019.0341

**Published:** 2020-02-10

**Authors:** Judit Salces-Ortiz, Carlos Vargas-Chavez, Lain Guio, Gabriel E. Rech, Josefa González

**Affiliations:** Institute of Evolutionary Biology (IBE), CSIC-Universitat Pompeu Fabra, Barcelona, Spain

**Keywords:** ATAC-seq, RNA-seq, cap-n-collar (cnc), adaptation, transcription factor-binding sites

## Abstract

Most of the genotype–phenotype analyses to date have largely centred attention on single nucleotide polymorphisms. However, transposable element (TE) insertions have arisen as a plausible addition to the study of the genotypic–phenotypic link because of to their role in genome function and evolution. In this work, we investigate the contribution of TE insertions to the regulation of gene expression in response to insecticides. We exposed four *Drosophila melanogaster* strains to malathion, a commonly used organophosphate insecticide. By combining information from different approaches, including RNA-seq and ATAC-seq, we found that TEs can contribute to the regulation of gene expression under insecticide exposure by rewiring *cis*-regulatory networks.

This article is part of a discussion meeting issue ‘Crossroads between transposons and gene regulation’.

## 1. Background

Understanding the link between genotype and phenotype is one of the major goals in evolutionary biology [1,2]. Even though substitutions of single nucleotide polymorphisms (SNPs) in the coding and regulatory regions of the genome can cause major changes on phenotypes [3,4], SNPs alone can only explain a fraction of the existing phenotypic variation [5–7]. Other types of mutations such as inversions, segmental duplications, transposable elements (TEs) and other structural variants, are also important sources of phenotypic variation [8–11]. Among these structural variants, TE insertions are likely to play a major role owing to their abundance and activity. TEs make up a sizeable proportion of virtually all genomes analysed to date [12–15]. Moreover, they have the ability to move around the genome generating a wide range of mutations during the process; from gene disruptions to variations in gene regulation [12,16]. Although most TE insertions are expected to be either deleterious or neutral, TEs have also been associated with fast and beneficial changes in phenotypes, fostering rapid adaptation [9,17,18].

There is increasing evidence on the functional role of some TE insertions [16,19–23]. One iconic example is the industrial melanism in the peppered moth. The dark form of the peppered moth, which rapidly increased in frequency in response to coal-polluted environments, is owing to a TE insertion in the first intron of the gene *cortex* [24]. However, we are still far from fully understanding the role TEs play in genotype–environment interactions. *Drosophila melanogaster* represents an excellent model for addressing this question. First, it has a small and well-annotated genome, which allows the putative genomic regions involved in the phenotypic changes to be more easily characterized [25]. Second, *D. melanogaster* has colonized many different environments in recent evolutionary time [26–28], demonstrating its ability to adapt very quickly, which has led to extensive phenotypic variation within and between populations [29]. Finally, *D. melanogaster* contains a wide repertoire of TE families, many of which contain polymorphic copies, suggesting that they are highly active [30–34]. Indeed, for some families, there is experimental evidence showing that they are active [35,36]. Moreover, several of these polymorphic TEs have been involved in phenotypic changes, probably linked to adaptive processes [37–44].

Besides works linking individual copies to phenotypic changes, an increasing number of investigations suggest that TEs have contributed to the rewiring of *cis*-regulatory networks during evolution, including pathways underlying processes such as pregnancy, brain development, innate immunity and stress response [17,45–49]. Owing to their repetitive and dynamic nature, TEs can distribute regulatory sequences such as promoters, transcription factor-binding sites and insulators across the genome altering gene expression [50,51]. In this context, it is reasonable to think that TEs can act as powerful agents to modify biological processes by creating *cis*-regulatory networks and rewiring already existing ones.

Pesticide resistance is an example of a rapid adaptive process that has resulted from a novel selective pressure [52]. In *Drosophila*, there is evidence to suggest that insecticides have played a large role in recent selection [53–58]. There are several examples of insecticide resistance in this species which has resulted from a TE affecting the expression of a relevant gene [38,40,43,44,59]. However, to date, to our knowledge, no genome-wide analysis of the potential role of TEs on the transcriptional response to insecticides has been performed.

In this work, we analysed gene expression profiles (RNA-seq), chromatin accessible regions (ATAC-seq), binding site predictions for the major transcriptional regulator of xenobiotic detoxification, cap-n-collar (cnc), [49,60] and signatures of selection in regions flanking TE insertions [33,49], in order to investigate the contribution of TEs to the regulation of gene expression in response to insecticide exposure. For this purpose, we exposed four *D. melanogaster* strains to malathion, a commonly used organophosphate insecticide (http://www.epa.gov). Our results suggest that TEs can contribute to the regulation of gene expression under insecticide exposure by rewiring *cis*-regulatory networks.

## 2. Methods

### (a) Fly stocks

Four *D. melanogaster* strains were used: SE_Sto_11_22 (*SE-Sto*), an isofemale strain collected in Stockholm, Sweden, in 2011 [61]; *RAL-375* and *RAL-177,* two strains from the Drosophila Genetic Reference Panel (DGRP) collected in Raleigh, North Carolina, USA [62], and the reference sequenced strain *iso-1* [63]. Flies were reared on fly food medium in a 12 : 12 h light/dark cycle at 25°C.

### (b) Xenobiotic exposure

To induce xenobiotic response, we used malathion, an organophosphate insecticide commonly used to control a variety of insects that attack fruits (http://www.epa.gov). Malathion was dissolved in 2-propanol and added to agar–sucrose medium to a final concentration of 20 µM. We used a unique dose for all strains as this is more similar to what flies experience in nature. For non-stress conditions, we used agar–sucrose food. We transferred the flies to new tubes with or without malathion and kept them for 9 h at 25 °C before gut dissection. Three biological replicates of 30 females each were performed for each condition and strain. No mortality was observed in any of the control replicates. After 9 h of malathion exposure, no mortality was observed in *SE-Sto* and *RAL-375*, while 15% and 25% mortality was observed in *RAL-177* and *iso-1*, respectively.

### (c) RNA isolation, library preparation and sequencing

Guts from 4- to 6-day-old females were dissected in 1× phosphate buffered saline (PBS) either under non-stress conditions or after xenobiotic exposure (stress). Total RNA was isolated using GenElute Mammalian genomic total RNA miniprep kit from SIGMA following the manufacturer's instructions. A 1.5 µg of total RNA from each sample was used for subsequent library preparation and sequencing. Briefly, library preparation was performed using the Truseq Stranded mRNA Sample Prep kit from Illumina following the manufacturer's instructions. Libraries were sequenced using Illumina 125 bp paired-end reads (25.4–57.8 million per sample, electronic supplementary material, table S1A).

### (d) Analysis of RNA-seq data

Quality of the fastq sequencing files was assessed using FastQC v. 0.11.8 (www.bioinformatics.babraham.ac.uk/projects/fastqc). TrimGalore v. 0.5.0 (www.bioinformatics.babraham.ac.uk/projects/trim_galore) was used for adapter contamination removal and Cutadapt v. 1.18 (default parameters) was used for low-quality trimming [64]. Trimmed reads were mapped to the *D. melanogaster* genome r6.15 using HISAT2 v. 2.1.0 [65]. On average, 93.3% of the reads were uniquely mapped to the genome. We explored technical duplications in our samples using dupRadar [66]. Overall, we found no bias towards high number of duplicates at low read counts, so we did not remove duplicates from the alignments. We used featureCounts v. 1.6.2 [67] for counting the number of reads mapping to genes for each sample (reverse-stranded parameter). The matrix of counting data was then imported into DESeq2 [68], an R bioconductor package, using the DESeqDataSetFromMatrix function. The DESeq main function, with default parameters, was used to obtain the library size-normalized read counts, which is used to identify differentially expressed genes (DEGs) and to generate the heat map and the principal component plots. DEGs were identified in pairwise comparisons modelling the samples as:∼strain + treatment + strain:treatment and adjusted using the Benjamini–Hochberg method to control for false discovery. The significant DEGs were identified after applying significance cut-offs (adjusted *p*-value ≤ 0.05 and fold-change ≥ 1.5). Note that DESeq2 normalization makes the expression of all genes comparable between samples, independently of sequencing depth and RNA composition [68].

### (e) Gene ontology analysis

Gene ontology (GO) enrichment analysis for biological process of the DEGs was performed using the functional analysis and clustering tool DAVID v. 6.8 (https://david.ncifcrf.gov) with default options [69,70]. All biological clusters above a score of 1.3 were considered as significantly enriched (electronic supplementary material, table S2) [70].

### (f) Generation of protein–protein interaction networks and identification of hub genes

Protein–protein interaction (PPI) networks were constructed with the DEGs of each strain using STRING v. 11.0 [71] taking into account experimental data, databases and gene co-expression data. The minimum required interaction score cut-off was set at 0.5. The PPI database generated by STRING was used in Cytoscape to rank nodes in the network by their network features. Hub genes (genes with a high number of edges) were also explored using the CytoHubba plugin r1.6 (http://hub.iis.sinica.edu.tw/cytoHubba/index.html) in Cytoscape. In this work, we first compared Degree (the most typically used methodology for PPI analysis) and Maximal Centrality Clique (MCC) algorithms to look for significant hub genes. According to the CytoHubba developer site (http://hub.iis.sinica.edu.tw/cytoHubba/supplementary/index.htm), Degree rank genes based on its number of interactions, while MCC captures proteins that are tightly connected to others and it is able to discover new feature nodes. The overlap between Degree and MCC methodologies in our work was between 78.4% and 100% when analysing the top 15% hub genes from each methodology. Thus, we used the MCC score to rank the top 15% hub genes from the four predicted PPI networks (electronic supplementary material, table S3A–D).

### (g) Transposable element dataset

The release 6 of the reference genome (*iso-1* strain) contains 5416 TE insertions [25]. We used *T-lex3* [72] to genotype these insertions in *SE-Sto*, *RAL-375* and *RAL-177* using whole-genome sequencing data [61,62]. Note that *T-lex3* cannot accurately estimate frequencies for TEs that are nested or part of segmental duplications (1552 TEs [33]) and failed to genotype another 65 insertions. Thus, we were able to genotype 3784 in at least one of the three strains (electronic supplementary material, table S4A). Most of these insertions, 78.6% (2975 out of 3784) were present in the four strains analysed, 11.9% (449 out of 3784) were only present in the reference genome (*iso-1*) and 9.5% (360 out of 3784) were polymorphic in the four strains (electronic supplementary material, table S4A).

### (h) DNA isolation for ATAC-seq

The protocol used for ATAC-seq was adapted from Buenrostro *et al.* [73] to be used for *D. melanogaster* tissue. Thirty guts from *Drosophila* females between 4 and 6 days old were isolated and immediately placed in 200 µl ice-cold PBS. Guts were lysed and homogenized to isolate between 50 000 and 100 000 cells using a Neubauer camera. The cells were washed and lysed to obtain a pellet with crude nuclei. The nuclei were resuspended in 47.5 µl of tagmentation buffer and incubated for 30 min at 37°C with 2.5 µl of Nextera Tn5 Transposase. DNA was purified using a Qiagen MinElute PCR Purification Kit. Finally, a library was generated using the Nextera kit and amplified by polymerase chain reaction. All libraries were sequenced using *Illumina* 50 bp paired-end reads (25 to 51.9 million per sample, electronic supplementary material, table S5A).

### (i) Analysis of ATAC-seq data

The general quality of the raw reads was evaluated using FastQC v. 0.11.8 (www.bioinformatics.babraham.ac.uk/projects/fastqc). The adapters were identified and removed from the reads using bbduk from the BBTools suite v. 38.00 (https://sourceforge.net/projects/bbmap/). Next, the reads were trimmed using Trimmomatic [74] with the following parameters: *LEADING:3 TRAILING:3 SLIDINGWINDOW:4:15 MINLEN:36*. The filtered reads were mapped against the *D. melanogaster* genome r6.15 using Bowtie v. 1.2.2 [75] with the following parameters: *-S -v 3 -a -m 100 –best –strata -X 2000 -p 10 –interleaved*. The output files were processed with CSEM v. 2.4 [76] and filtered using *piPipes_bam_ZW_filter* from piPipes [77]. Finally, only pairs of reads with an insert size smaller or equal to 100 bp were conserved.

The peaks were predicted following the strategy described in the ENCODE ATAC-seq pipeline (https://github.com/ENCODE-DCC/atac-seq-pipeline). The *bam* files were first deduplicated. Next, they were converted into *tagAlign* (*bed*) format. The *tagAlign* files for the three replicates for each strain under the same conditions were merged and then split into two sets randomly. Peaks were predicted in each of the two sets using MACS2 v. 2.1.2 [78] with the following parameters: *-p .01 –shift 75 –extsize 150 –nomodel -B –SPMR –keep-dup all –call-summits*. Only peaks with an overlap greater than 50% between both sets of predicted peaks were conserved. For this set of peaks, the irreproducible discovery rate (IDR) was calculated and only peaks with an IDR less than 0.05 were conserved. The peaks were rescaled around the predicted summits by extending 100 bp to both sides of the summit. Finally, the predictions for all strains in both conditions were merged into a single-bed file comprising the universal set of peaks. To define whether a peak was open or not, the background noise was calculated generating windows spread randomly throughout the genome and calculating the median of the coverage in each window. If the median coverage in a peak was above the background noise, it was considered to be open.

### (j) Expression analysis of transposable element families

For both mapping and analysing the expression of TEs, we used the TEtools pipeline [79]. Briefly, data were trimmed using UrQt [80] in order to remove low-quality nucleotides. The resulting trimmed reads were aligned to a TE library using Bowtie2 v. 2.3.4.1 [81]. The TE library was created from the TE family information obtained from Repbase. The read count step was computed per TE family, adding all reads mapped on copies of the same family. Finally, we performed the differential expression analysis between TE families using the R Bioconductor package DESeq2 [68] on the raw read counts retaining the TE families with a *p*-value ≤ 0.05 and fold-change ≥ 3.

## 3. Results

### (a) Strains tolerant to malathion showed a lower number of differentially expressed genes compared with sensitive strains

**Figure 1.**
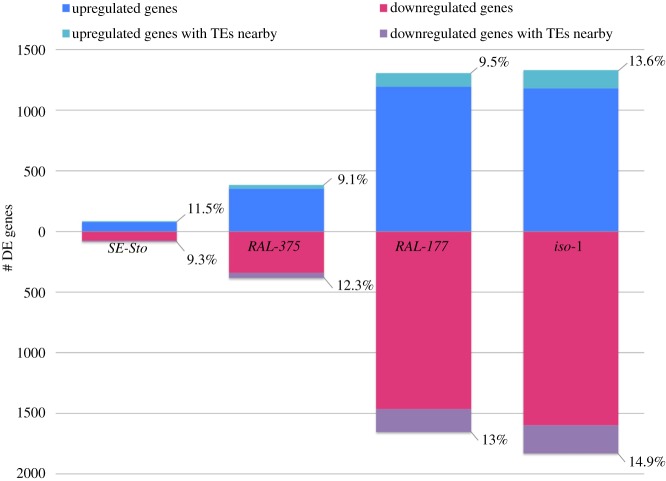
Number of DEGs in response to malathion. DEGs in the *SE-Sto* strain collected in Stockholm (Sweden), two North American strains from the DGRP panel, *RAL-375* and *RAL-177,* and the sequenced strain *iso-1*. The percentage of DEGs with TEs nearby per strain is also shown. Note that while no mortality after 9 h of malathion exposure was found for *SE-Sto* and *RAL-375*, 15% and 25% mortality was found for *RAL-177* and *iso-1*, respectively.

To investigate the effect that malathion has on gene expression and the potential role of TEs in these changes, we performed RNA-seq analysis on four *D. melanogaster* strains that differed in their tolerance to malathion. After 9 h of malathion exposure, no mortality was observed in *SE-Sto* and *RAL-375*, while 15% and 25% mortality was observed in *RAL-177* and *iso-1*, respectively. Overall, the number of DEGs ranged from 153 to 2778, similar to other transcriptomic studies performed with xenobiotics (figure 1 and table 1; electronic supplementary material, table S1B–E) [58,82]. Among these DEGs, we found several xenobiotic-related genes including *Cyp12d1-d, Cyp6g1*, *Jheh1* and *Jheh2,* that have previously been identified as major malathion resistance loci (electronic supplementary material, table S1F) [83].

**Table 1.** Number of DEGs in response to malathion exposure per strain, and number of DEGs shared among strains. (Percentages in parentheses correspond to the strain in the column.)

no. DEGs	*SE-Sto*	*RAL-375*	*RAL-177*	*iso-1*
*SE-Sto*	153			
*RAL-375*	17 (11.1%)	694		
*RAL-177*	40 (26.1%)	311 (44.8%)	2659	
*iso-1*	78 (51%)	304 (43.8%)	1096 (41.2%)	2778

The number of DEGs was lower in the more tolerant strains compared with the more sensitive ones (figure 1 and table 1). Fifty-one per cent of the DEGs in the *SE-Sto* tolerant strain were also found to be differentially expressed in the *iso-1* sensitive strain (table 1). However, the majority of these genes (75 of 78) showed opposite expression patterns, consistent with the different phenotyping profiles of these strains (electronic supplementary material, figure S1). These 75 genes were enriched for ‘cell cycle’ biological processes (electronic supplementary material, table S2A). Moreover, the most sensitive strains (*RAL-177* and *iso-1*) shared 41.2% of the DEGs and most of them (1054 of 1096) showed the same pattern of expression, which probably reflects that both strains are highly stressed and struggling to survive (table 1; electronic supplementary material, figure S1). Indeed, the shared upregulated DEGs are mainly related with ‘cellular response to stimulus', ‘cellular transport’ and several ‘metabolic processes’, while the shared downregulated DEGs have mostly functions related with ‘circadian rhythm’, ‘ageing’ or ‘molting’ (electronic supplementary material, table S2B).

### (b) Only metabolic and stress response genes are upregulated after malathion exposure in the most tolerant strains

**Figure 2.**
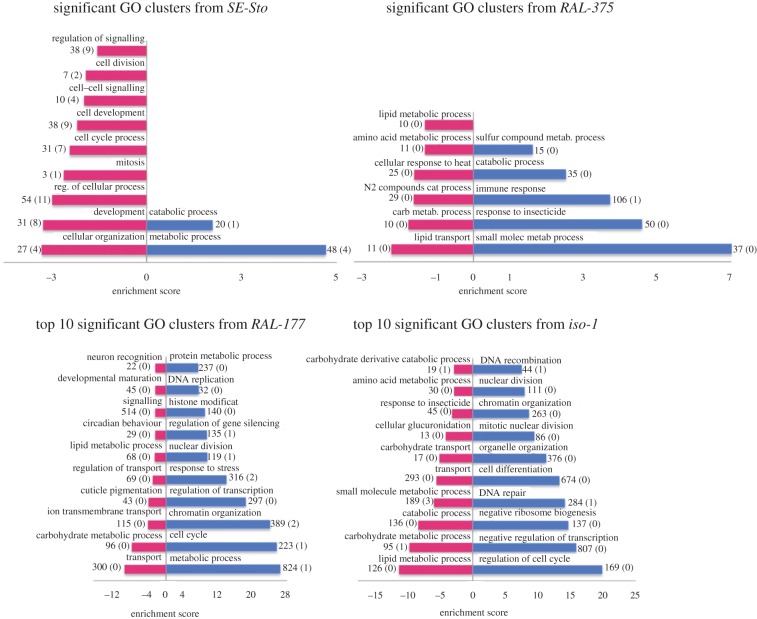
Significant gene ontology (GO) clusters according to DAVID functional annotation tool for upregulated (blue) and downregulated (red) genes. For strains with more than 10 significant GO clusters, only the top 10 are shown (enrichment score greater than 1.3). The horizontal axis represents the enrichment score. For each cluster, the number of DEGs and the number of DEGs with TEs nearby (in parentheses) are given.

Besides analysing the GO enrichment of DEGs shared between strains, we also analysed each strain individually. In the *SE-Sto*, only ‘metabolic processes’ were upregulated, while in the *RAL-375*, ‘response to insecticide’ was also upregulated, suggesting that this strain was more affected by stress (figure 2; electronic supplementary material, table S2C,D). In both cases, the upregulation of metabolic-related genes suggests the potential detoxification ability via metabolic processes of these strains [58]. In *RAL-177*, besides ‘response to stress’ and ‘metabolism’, ‘cell cycle’, ‘chromatin organization’ and ‘nuclear division’ were also upregulated (figure 2; electronic supplementary material, table S2E). In addition to these, ‘negative regulation of transcription’ and ‘negative ribosome biogenesis' are upregulated in the *iso-1* strain, suggesting that this strain is entering apoptosis (electronic supplementary material, table S2F). Finally, the number of downregulated genes involved in ‘lipid and carbohydrate metabolism’ was higher in sensitive strains as previously reported by Riahi *et al.* [58] (figure 2; electronic supplementary material, table S2E,F). Overall, the patterns of GO enrichment were in agreement with the differences in malathion tolerance observed across strains.

### (c) Up to 14.4% of differentially expressed genes are located nearby transposable elements

To identify the role that TEs could have in response to malathion, we analysed the proportion of DEGs that were located nearby annotated TEs (less than 1 kb or inside the gene). For the *iso-1* strain, we analysed all the annotated TEs (5416 TEs [25]) and for the other three strains, we analysed the TEs that could be genotyped using *T-lex3* [72] (3741 in *SE-Sto*, 3637 in *RAL-375* and 3651 in *RAL-177*, electronic supplementary material, table S4A, see Material and methods). We found that up to 14.4% of DEGs are located nearby annotated TEs (figure 1; electronic supplementary material, table S4B). DEGs were enriched nearby TE insertions compared with the overall distribution of genes nearby TEs in the genome for the four strains tested, although this enrichment was only significant in *RAL-177* and *iso-1* (*χ*^2^ test, *p*-value = 0.00019 and 0.005294, respectively; electronic supplementary material, table S4B). Among those TEs found in proximity to DEGs, we found *FBti0019430*, *FBti0018880* and *FBti0019627*, which have been shown to play a role in insecticide resistance [38,40,43,84].

**Figure 3.**
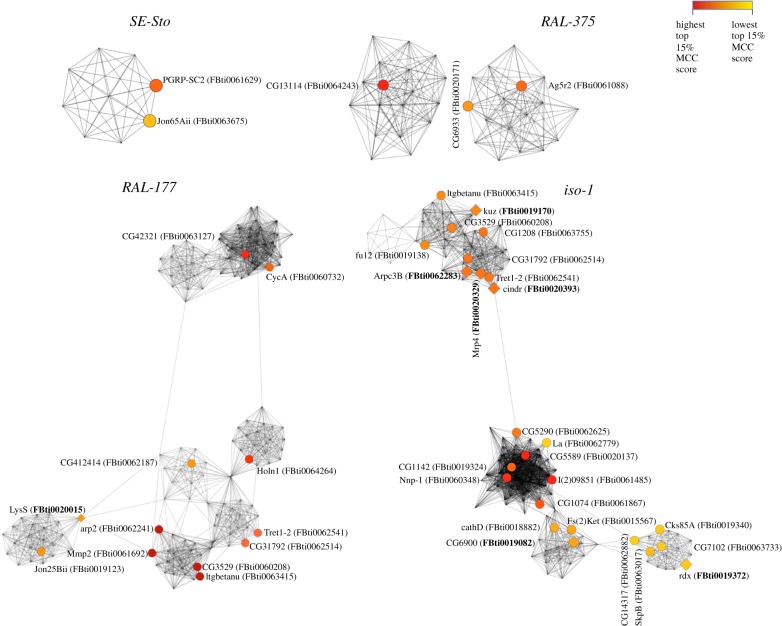
PPI networks per strain are shown for the DEGs with the top 15% MCC scores. Colour gradient indicates the MCC values: from higher MCC scores (red) to lower MCC scores (yellow). Circular nodes represent genes with TEs nearby, while diamond-shaped nodes represent genes with TEs nearby with evidence of selection (in bold) [33,49]. Note that four hub genes nearby TEs are present both in *RAL-177* and in *iso-1*.

To identify those TEs that are likely to have a greater effect in the response to malathion exposure, we used DEGs to construct PPI networks. We focused on the top 15% of genes according to the MCC score, which captures proteins that are tightly connected to other proteins. Only 76 of the 307 unique hub genes (approx. 25%) have previously been identified as xenobiotic stress response candidates (electronic supplementary material, table S3E). We found that 38 of the hub genes have TEs nearby (electronic supplementary material, table S3E). In most cases, these genes are within the ones with the highest MCC values (figure 3). Moreover, some of the hub genes that connect subnetworks are located nearby TEs, such as *LysS, arp2* and *Mmp2* in *RAL-177* (figure 3). Thirty of these hub genes have a single TE nearby, which could be considered the most likely candidate to affect their expression (electronic supplementary material, table S3F). Indeed, seven of these 30 TEs have previously been identified as showing signatures of selection, and most of them have been related to stress response (table 2; electronic supplementary material, table S3F) [33,49]. We checked whether any of the 17 TEs located nearby upregulated hub genes contained binding sites for cnc, the major transcription factor in xenobiotic and oxidative stress response [60]. We found that five TEs contained cnc-binding sites, including three TEs with evidence of selection (table 2; electronic supplementary material, table S3F) [33,49]. Overall, we identified 30 TEs located nearby hub DEGs and thus, likely to have a bigger effect in malathion response. Nine of these TEs have evidence of selection and/or cnc-binding sites (table 2).

**Table 2.** Description of the TE insertions located nearby differentially expressed hub genes and having either evidence of selection, cnc-binding sites (BS), or ATAC-seq peaks.

candidate TEs	TE family	nearby gene	TE location	gene product/stress related	evidence of selection [33,49]	cnc BS	ATAC-seq peaks
*FBti0019170*	*F-element*	*kuz*	inside gene	metalloendopeptidase/zinc tolerance related [85]	fTE	0	—
*FBti0019372*	*S-element*	*rdx*	inside gene	ubiquitin ligase binding protein/-	H12	1	—
*FBti0019082*	*Rt1b*	*CR6900*	5′ of gene	pseudogene /-	TajimaD	3	yes
*FBti0020393*	*1360*	*cindr*	Inside gene	adaptor protein/-	TajimaD	0	—
*FBti0020329*	*G5*	*Mrp4*	5′ of gene	ABC transporter/response to oxidative stress [86]	TajimaD	0	—
*FBti0062283*	*ninja-Dsim-like*	*Arpc3B*	5′ of gene	actin-related protein 2/3 complex/cold tolerance and bacterial infection	TajimaD	0	—
*FBti0020015*	*412*	*LysS*	5′ of gene	lysozyme/immune response [87,88]	young and long	3	—
*FBti0015567*	*Tirant*	*Fs(2)Ket*	inside gene	nuclear protein import/-	—	1	—
*FBti0020137*	*S-element*	*CG5589*	3′ of gene	adenosintriphosphate/-	—	1	—
*FBti0062779*	*1360*	*La*	5′ of gene	la autoantigen-like/-	—	0	yes
*FBti0061088*	*INE-1*	*Ag5r2*	5′ of gene	antigen 5-related 2/-	—	0	yes
*FBti0064264*	*INE-1*	*Holn1*	3′ of gene	U5 small nuclear ribonucleoprotein particle/wound response [89]	—	0	yes

### (d) Most of the differentially expressed genes have ATAC-seq peaks

**Figure 4.**
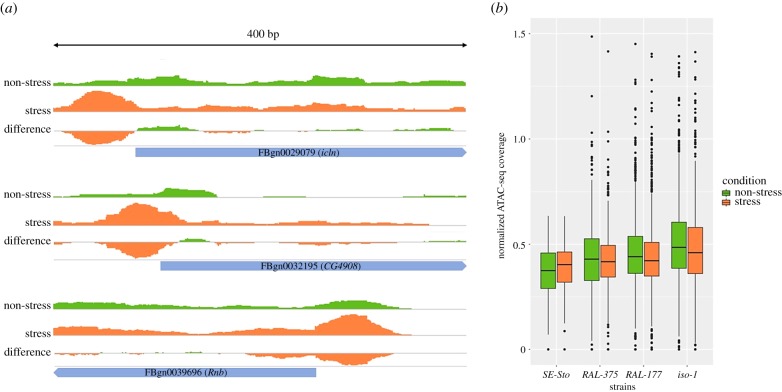
Changes in chromatin accessibility after malathion exposure. (*a*) Changes in the chromatin accessibility at the promoter region of three individual upregulated genes. (*b*) Global ATAC-seq coverage in all upregulated genes in each strain. The values are scaled, so that the median values of the first and 20th quantiles correspond to 0 and 1.

We analysed the chromatin accessibility genome-wide in non-stress and stress conditions. The total number of ATAC-seq peaks identified was similar among strains, 23 562 on average, and comparable with the previous studies (electronic supplementary material, table S5B) [90–92]. As expected, most of the peaks were located in gene bodies or promoter regions (approx. 92.2%) (electronic supplementary material, table S5B) [90]. If we focus on DEGs, approximately 83.6% of them had peaks assigned to their gene bodies or promoter regions. Although some upregulated genes showed increased accessibility in stress compared with non-stress conditions, most of the genes did not, as has been previously reported (figure 4; electronic supplementary material, table S5C) [93]. Similarly, most downregulated genes did not show decreased accessibility in stress versus non-stress conditions (electronic supplementary material, table S5C). Finally, we also tested whether peaks located nearby upregulated genes had cnc motifs. We found that, on average, approximately 15% of the peaks contained cnc motifs except in *SE-Sto*, where this percentage was 6.5%. This lower percentage in the *SE-Sto* strain is consistent with the lower number of DEGs and the lack of mortality, suggesting that this strain is experiencing lower levels of stress.

### (e) Half of the transposable elements located in open chromatin regions are distal to genes

We identified 199 TEs across strains that overlap with ATAC-seq peaks (electronic supplementary material, table S5D). Most of these TEs showed peaks in both non-stress and stress conditions (74%). Still, on average, approximately 19.1% were only present in non-stress conditions and approximately 22.6% were only present in stress conditions. Ninety-five of these TEs were located inside genes or in promoter regions, and 30 of them were located inside DEGs or in their promoter regions (electronic supplementary material, table S5E). These 30 TEs are enriched for *1360* family (electronic supplementary material, table S5E). Three of these 30 TEs have evidence of selection (*FBti0019430*, *FBti0060443* and *FBti0019082*), two contain cnc-binding sites (*FBti0061018* and *FBti0062187*) and three are located in the promoter or inside hub genes (the aforementioned *FBti0019082* and *FBti0061088*, and *FBti0062779*) (table 2).

However, the other 104 TEs with peaks are distal to genes. These TEs are enriched for several families including *Rt1b* and *gypsy8* (electronic supplementary material, table S5D). Two of the 104 TEs have evidence of selection (*FBti0019419* and *FBti0019355*) and nine contained a cnc motif (electronic supplementary material, table S5F). Thus, our results suggest that half of the TEs located in open chromatin are distal to genes.

### (f) Fifty transposable element families showed differences in expression after malathion exposure

**Figure 5.**
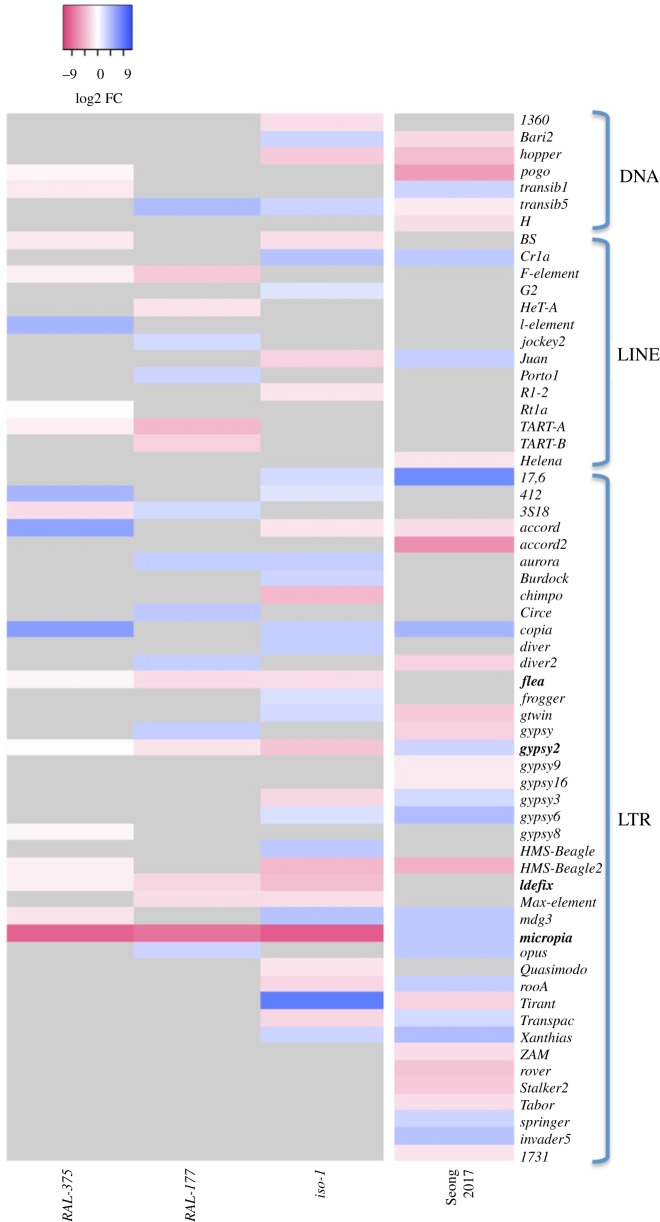
Heat map showing the differentially expressed TE families after malathion exposure. On the left, the three strains with differentially expressed TEs from our work. In bold, the TE families shared among the strains. Four families, *flea, gypsy-2, Idefix* and *micropia*, were differentially expressed in the three strains, 12 in two strains and 34 only in one strain. On the right, the expression profile of TE families using data from Seong *et al*. [54].

Finally, we also checked whether the expression of TE families was affected by the malathion treatment. While no differentially expressed TE families were detected in *SE-Sto*, 50 families were differentially expressed between non-stress and stress conditions in at least one of the other three strains (figure 5; electronic supplementary material, table S6A). The pattern of expression was family dependent as has been previously suggested (figure 5) [94]. Similar to the differences in the number of DEGs across strains, we observed that the most tolerant strains have a lower number of differentially expressed TE families compared with the most sensitive strains (Pearson correlation *r* = 0.8).

Both upregulated and downregulated TE families were enriched for long terminal repeat (LTR) elements when compared with all the TEs annotated in the genome (*χ*^2^, *p*-value = 1.78 × 10^−30^ and 8.26 × 10^−25^ for upregulated and downregulated, respectively).

We checked whether copies from these differentially expressed TE families were located nearby DEGs. We found that *diver2, Bari1* and *accord* copies were mostly located nearby upregulated genes (electronic supplementary material, table S6B). Thus, we cannot discard that the upregulation observed for these three families was owing to the upregulation of genes located nearby.

Finally, we also analysed an RNA-seq dataset obtained from two *D. melanogaster* strains that differed in their dichlorodiphenyltrichloroethane tolerance [54]. We identified 36 differentially expressed TE families, 24 of them overlapped with our dataset of differentially expressed families (figure 5). However, this overlap is not statistically significant (*χ*^2^ test, *p*-value = 0.3314), and most of the observed changes were in the opposite direction (electronic supplementary material, table S6C).

## 4. Discussion

In this work, we analysed the gene expression profiles (RNA-seq), the chromatin accessible regions (ATAC-seq), cnc-binding site predictions [49,60] and signatures of selection in regions flanking TE insertions [33,49], to identify TE insertions likely to contribute to the genomic response to malathion. Our analysis of four *D. melanogaster* strains has shown that the number, the pattern of expression (up- or downregulation) and the GO enrichment of DEGs in response to malathion were consistent with the differences among strains in their tolerance level to this insecticide (figures 1 and 2; electronic supplementary material, figure S1). Our results are consistent with a recent analysis of a mutant *D. melanogaster* strain sensitive to xenobiotics in which the number of DEGs was also higher than in the most tolerant control strain [58]. While similar results have been found in the green peach aphid [95], more DEGs were found in the most resistant strains of *Aedes Aegypti* mosquitoes [96].

Our results also suggest that the control of energy consumption is relevant to stress response, as the number of downregulated genes involved in lipid and carbohydrate metabolism was higher in sensitive strains [58]. Finally, while we found that several of the DEGs have been previously identified as candidates for xenobiotic response, including four of the five major genes, we failed to identify others. However, it is known that the expression of stress response genes is time dependent, with genes not actively expressed along the entire stress period [83,97–99].

We identified 38 hub genes located nearby TE insertions. However, only three of these TEs had an ATAC-seq peak and were located in the promoter or inside the hub gene. One possible explanation is that some TEs are bound by numerous transcription factors and other co-activators that could prevent the Tn5 transposase from cutting in these regions [100,101]. Indeed, for some stress genes, it has been shown that *RNA polymerase II* is associated with their promoter regions prior to the induction of the stress [102]. In addition, the number of TEs with identifiable peaks might be underestimated. Given the repetitive nature of the TEs, there are limitations when attempting to accurately map the reads to their correct position. While using CSEM [76] increases the number of uniquely mapping reads and thus reveals additional peaks, the remaining discarded multi-mapping reads might have arisen from TEs located in regions with open chromatin. Combining ATAC-seq with histone mark information could further inform on the potential role of TEs as enhancers and promoters, although it has been shown that this is not always the case [49,103].

If we consider those TEs that are located nearby differentially expressed hub genes and have either ATAC-seq peaks, evidence of selection and/or cnc-binding sites, we identified a dataset of 12 insertions, seven of them located nearby genes not previously related to stress response (table 2). However, *rdx* regulates the *Hedgehog* signalling pathway involved in cell survival under stress conditions [104], and *cindr* is a multi-adaptor protein that has been related to the activation of the p38 pathway in response to oxidative stress [105,106]. Thus, our results suggest that TEs located nearby these genes could play an important role in xenobiotic stress response. Although there is not a clear association between the presence/absence of these TE insertions and the change in expression of their nearby genes (electronic supplementary material, table S4C), this result is consistent with previous analysis showing that the effect of TEs on the expression of nearby genes is background dependent [39,94]. More genetic backgrounds should be analysed to elucidate whether TEs have a specific effect restricted to particular backgrounds or whether the effect of TEs is more general.

## 5. Conclusion

We found that TEs can contribute to the genome-wide response to insecticide resistance as suggested by the association of TEs with differentially expressed hub genes. Other TEs identified in this work can also influence insecticide response, as exemplified by *FBti0019430*, *FBti0018880* and *FBti0019627* that have been previously reported to be involved in insecticide response [38,40,43,84]. Our results also suggest that the effect of TEs on gene expression in response to insecticides is background dependent. Functional validation of the candidate TEs in several backgrounds would help determine whether the effect of TEs in response to insecticide is global or restricted to particular backgrounds.

## Supplementary Material

Figure S1. Differentially expressed genes shared among strains

## Supplementary Material

Table S1. Differentially gene expression results

## Supplementary Material

Table S2. Gene ontology enrichment analysis

## Supplementary Material

Table S3. Identification of hub genes in the four strains analyzed in this work

## Supplementary Material

Table S4. Genotyping and genome distribution of TEs for each analyzed strain

## Supplementary Material

Table S5. ATAC-seq analysis

## Supplementary Material

Table S6. TE family expression analysis

## Data accessibility

Sequencing data are deposited at SRA with the accession number PRJNA551114. The additional datasets supporting this article have been uploaded as part of the supplementary material.
