# Supplementary material for: Transposable elements contribute to the genomic response to insecticides in Drosophila melanogaster
Source: Philos Trans R Soc Lond B Biol Sci. 2020 Feb 10;375(1795):20190341. doi: 10.1098/rstb.2019.0341 (PMC7061994; doi:10.1098/rstb.2019.0341)

Supplementary figure 1. Venn diagrams of differentially expressed genes shared among strains. A). Venn diagram of all DEGs per strain. B). Venn diagram from all up-regulated genes per strain. C). Venn diagram from all down-regulated genes per strain. From 78 DEGs shared between *SE-Sto* and *iso-1* (A), only 3 are up-regulated in the two strains (B) and none is down-regulated in the two strains (C). Thus, genes shared between these two strains show opposite expression patterns.

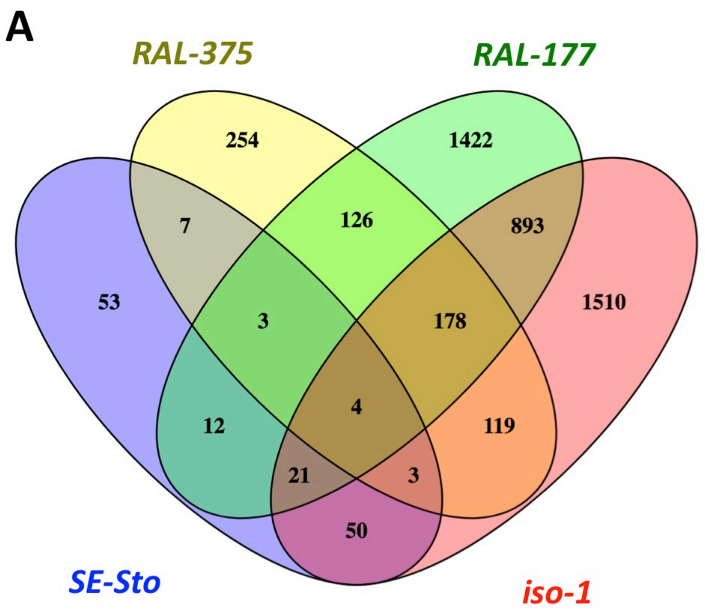

Supplement: Figure S1. Differentially expressed genes shared among strains [file rstb20190341supp1.pdf]
